# Survey on Awareness and Attitudes Toward Maternal Immunization Against Influenza, Pertussis, Respiratory Syncytial Virus, and Group B Streptococcus Among Pregnant Women in Japan

**DOI:** 10.3390/vaccines13080779

**Published:** 2025-07-23

**Authors:** Kazuya Hiiragi, Soichiro Obata, Masafumi Yamamoto, Mai Shimura, Chika Akamatsu, Azusa Tochio, Mayumi Hagiwara, Aya Mochimaru, Ai Kiyose, Miki Tanoshima, Etsuko Miyagi, Shigeru Aoki

**Affiliations:** 1 Perinatal Center for Maternity and Neonates, Yokohama City University Medical Center, Yokohama 232-0024, Japan; khiiragi@yokohama-cu.ac.jp (K.H.); mikitano@yokohama-cu.ac.jp (M.T.);; 2 Department of Obstetrics and Gynecology, Yokosuka Kyosai Hospital, Yokosuka 238-8558, Japan; 3 Department of Obstetrics and Gynecology, Yokohama Rosai Hospital, Yokohama 222-0036, Japan; 4 Department of Obstetrics and Gynecology, Yokohama City University Hospital, Yokohama 236-0004, Japanemiyagi@yokohama-cu.ac.jp (E.M.); 5 Department of Obstetrics and Gynecology, National Hospital Organization Yokohama Medical Center, Yokohama 245-0063, Japan; 6 Department of Obstetrics and Gynecology, Saiseikai Yokohama-shi Nanbu Hospital, Yokohama 234-0054, Japan; 7 Department of Obstetrics and Gynecology, Fujisawa City Hospital, Fujisawa, Kanagawa 251-8550, Japan; 8 Department of Obstetrics and Gynecology, Yokohama Minami Kyosai Hospital, Yokohama 236-0037, Japan

**Keywords:** vaccine, pregnancy, pertussis, influenza, respiratory syncytial virus, group B streptococcus, maternal immunization

## Abstract

**Background/Objective**: Maternal immunization is highly recommended, particularly in developed countries. However, its awareness among pregnant women in Japan remains low. This study aimed to assess the awareness and attitudes toward maternal immunization among pregnant women in Japan and to identify the factors that may promote its acceptance. **Methods**: We conducted a cross-sectional questionnaire survey among pregnant women attending antenatal checkups at nine facilities in Kanagawa Prefecture, Japan, from August 2024 to January 2025. The survey assessed knowledge and intention regarding maternal immunization for influenza, pertussis, respiratory syncytial virus (RSV), and group B streptococcus (GBS) as well as attitudes toward vaccination costs and information sources. **Results**: Overall, 523 respondents were included in this study. The overall awareness of maternal immunization was 16%. Willingness to receive vaccinations during pregnancy was reported for influenza (68%), pertussis (58%), RSV (59%), and GBS (71%). A common reason for vaccine hesitancy included uncertainty about its effects on the fetus. The key factors associated with vaccine acceptance were higher educational attainment and prior knowledge of maternal immunization. Regarding costs, most respondents were willing to pay up to JPY 5000 (approximately USD 35). The most frequently prioritized sources were explanations from physicians, followed by explanations from midwives. **Conclusions**: Despite low awareness, vaccination intention was comparable to that reported in other countries. Points that may contribute to improved vaccine uptake were also identified. These findings may lead to the prevention of infectious diseases in newborns and infants in Japan and possibly improve public health.

## 1. Introduction

Maternal infection during pregnancy is a known contributor to serious maternal illness. Since immune responses in early infancy remain immature and largely rely on maternally derived antibodies, maternal immunization, which promotes the transplacental transfer of protective antibodies, plays an essential role in safeguarding both maternal and neonatal health [1]. Accordingly, maternal immunization is actively recommended and widely implemented in countries, such as the United States [2,3]. Currently, the CDC and WHO recommend the administration of vaccines against COVID-19, influenza, respiratory syncytial virus (RSV), and tetanus, diphtheria, and acellular pertussis (Tdap) during pregnancy [4,5]. Conversely, although seasonal influenza vaccination during pregnancy is common in Japan, the administration of pertussis-containing vaccines to pregnant women is hardly performed, partly due to the absence of strong recommendations in clinical guidelines, a situation similarly observed for COVID-19 and RSV vaccines [6,7]. Consequently, awareness of maternal immunization among pregnant women in Japan remains low. A previous study conducted in 2019 reported a recognition rate of only 3.9% [8], which may reflect the absence of Tdap approval, financial burden, and insufficient recommendations from healthcare providers [7]. In 2024, a vaccine against RSV was approved for pregnant women and became available in Japan. Currently, several maternal immunization vaccines, including those under development for the prevention of group B streptococcus (GBS), a major cause of severe infections during neonatal and early infancy, are being developed [9,10]. These developments highlight the growing significance of maternal immunization.

This study aimed to assess the awareness and attitudes toward maternal immunization among pregnant women in Japan and identify factors that may promote its acceptance.

## 2. Materials and Methods

### 2.1. Study Design and Participants

This multicenter cross-sectional study was conducted at two university hospitals, six general hospitals, and one obstetric clinic in Kanagawa Prefecture, Japan. Pregnant women attending routine antenatal checkups at any of these facilities were invited to participate in this study. Pregnant women who provided informed consent were enrolled between August 2024 and January 2025. The survey was conducted anonymously using Google Forms. Consent for participation was obtained from participants at the beginning of the questionnaire. Pregnant women who declined to provide consent were excluded.

The study protocol was approved by the Ethics Committee of Yokohama City University Medical Center Hospital (approval no. IPPAN 2024-019) and was conducted in accordance with the Declaration of Helsinki.

### 2.2. Data Collection

Participants were provided with a simplified explanation sheet about maternal immunization as well as specific information on pertussis, RSV, and GBS. Subsequently, participants scanned the QR code attached to the sheet, accessed the questionnaire page, and answered the questionnaire. Consent was obtained upon submission of responses. For questions related to GBS vaccination, a note clarified that the vaccine was assumed to be safe and effective for both mothers and infants.

The questionnaire included items on demographic and obstetric characteristics, disease awareness, vaccination intentions, and acceptable costs. Some items allowed the respondents to skip questions. The full questionnaire is provided in the Supplementary File. No skip logic was implemented; all participants were presented with the same set of questions regardless of their previous answers. This study was exploratory in nature, aimed at understanding current awareness and attitudes toward maternal immunization to inform clinical promotion strategies. Therefore, the questionnaire items were developed de novo by the study team rather than adapted from pre-validated instruments.

### 2.3. Statistical Analyses

Categorical data are reported as frequencies (percentages), and continuous data are provided as medians and interquartile ranges. Missing data were excluded from each relevant analysis and not imputed.

Statistical analysis was performed using the chi-square test for categorical variables and the Wilcoxon test for continuous ones. Logistic regression analyses were performed in a univariate manner; each variable was evaluated separately without adjustment for potential confounders. All variables collected in the questionnaire were tested individually, without preselection, to explore their associations with vaccination intention. Odds Ratios (ORs) and 95% Confidence Intervals (CIs) were determined. Differences were considered statistically significant at *p* < 0.05. Data were analyzed using JMP Pro 17 software (version 17.0.0; SAS Institute Inc., Cary, NC, USA).

## 3. Results

Overall, 523 valid responses were obtained. The participant characteristics are shown in Table 1. The overall awareness of maternal immunization was 16%. Moreover, the proportion of respondents who expressed willingness to receive vaccination during pregnancy was 68%, 58%, 59%, and 71% for influenza, pertussis, RSV, and GBS, respectively, with statistically significant differences among the vaccines (*p* < 0.001). The intention rate for GBS vaccination was assessed under the hypothetical assumption that the vaccine would be safe and effective. These proportions were calculated using only the number of respondents who answered each respective item: 521 for influenza (352/521), 518 for pertussis (303/518), 518 for RSV (305/518), and 519 for GBS (366/519). Summaries of response counts and missing data for each vaccine item are provided in Supplementary Table S1.

**Figure 1:**
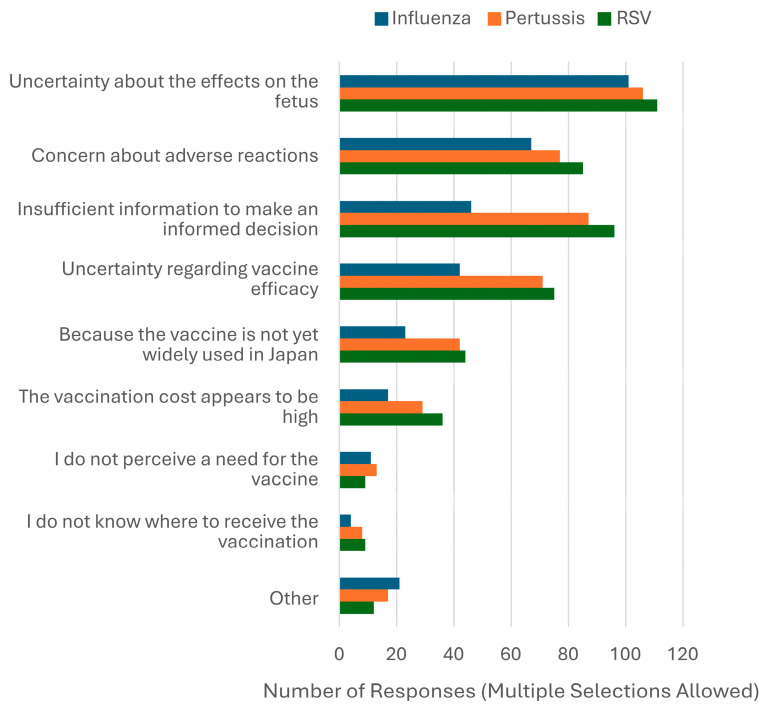
Reported reasons for unwillingness to receive maternal vaccines.

**Figure 2:**
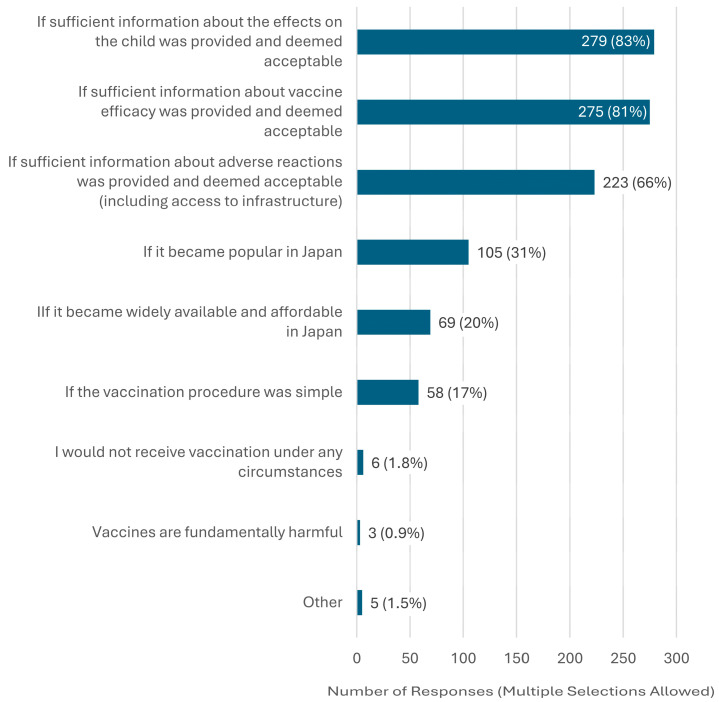
Conditions under which pregnant women would consider GBS vaccination (n = 338). Note: n = 338 refers to all participants, regardless of their initial willingness to receive the GBS vaccine, who answered the question about conditions under which they would consider future GBS vaccination. Multiple responses were allowed.

The most common reason for unwillingness to receive vaccination was “Uncertainty about the effects of influenza, pertussis, and RSV on the fetus.” Other frequently cited reasons included concerns about adverse reactions and insufficient information to make an informed decision (Figure 1). Participants could select multiple reasons. Detailed counts and percentages of reasons for vaccine hesitancy, categorized by vaccine (influenza, pertussis, or RSV), are presented in Supplementary Table S2. Regarding GBS, the most frequently cited condition under which respondents would consider vaccination was “If sufficient information about the effects on the child was provided and deemed acceptable” (Figure 2). The GBS vaccine remains under development and has not yet received regulatory approval in Japan or any other country. Accordingly, the survey did not assess vaccine hesitancy per se but rather explored respondents’ willingness to receive the vaccine in the future and the conditions under which they would consider doing so.

Regarding costs, among those who were willing to be vaccinated, the amount most frequently selected as the upper limit for vaccination was under JPY 3000. There were no differences between the vaccines, and most respondents were willing to pay up to JPY 5000. The proportion of respondents who answered that they would not receive vaccination regardless of the cost was 7.8%, 8.6%, 7.8%, and 6.9% for influenza, pertussis, RSV, and GBS, respectively (Table 2).

**Figure 3:**
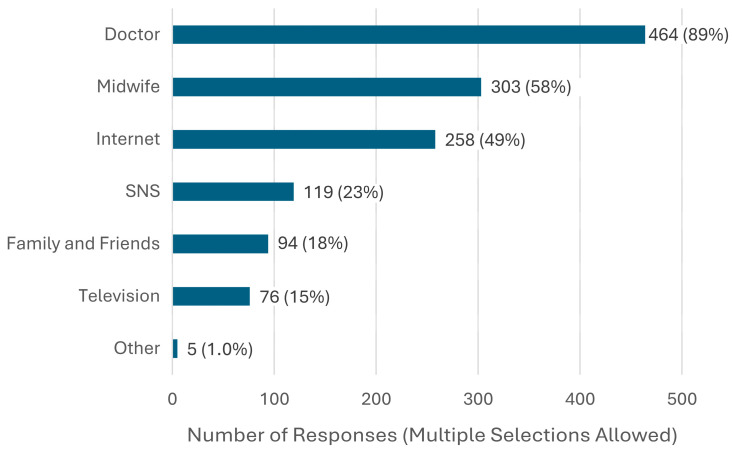
Key sources of information on vaccines during pregnancy (n = 522).

When obtaining information about vaccination during pregnancy, the most frequently prioritized sources were explanations from doctors, followed by explanations from midwives (Figure 3). In the analysis of factors associated with willingness to be vaccinated, factors commonly observed across all vaccines were final educational attainment (junior college, vocational school, or technical college graduate or higher) and knowledge of maternal immunization. Additionally, occupation was positively associated with vaccine acceptance for influenza, RSV, and GBS. Pregnant women who identified physicians or midwives as key sources of information were highly likely to accept influenza and pertussis vaccines. Disease awareness was also significantly associated with vaccine acceptance for influenza (OR 2.70, 95% CI: 1.04–6.61), RSV (OR 1.75, 95% CI: 1.16–2.64), and GBS (OR 2.13, 95% CI: 1.31–3.45). A history of RSV infection in the respondent or their child was frequently observed among those who were willing to be vaccinated (OR 1.70, 95% CI: 1.08–2.68). Finally, gestational age was significantly associated with vaccine acceptance only for GBS (Table 3, Table 4, Table 5 and Table 6). A summary of variables that showed statistically significant associations with willingness to receive each vaccine is presented in Supplementary Table S3.

## 4. Discussion

This study showed that the awareness rate of maternal immunization among pregnant women in Japan was 16% and the intention to receive the vaccination was approximately 60%. In addition, factors associated with increased vaccination rates were prior knowledge of maternal immunization and the cost burden of vaccinations.

Although awareness of maternal immunization remains low in Japan compared to other countries, where more than half of pregnant women are reportedly aware of maternal immunization and willing to receive maternal vaccines [11,12,13], the intention to vaccinate observed in this study was comparable. For RSV, the most recent maternal immunization, international studies have reported intention rates ranging from 42% to 88% [14], which aligns with the rate found in our study. In Japan, awareness appears to be gradually increasing: the proportion of pregnant women aware of maternal pertussis vaccination rose from 3.9% in 2019 [8] to 16% in this study. This discrepancy between low awareness and relatively high intention may be interpreted through the WHO BeSD framework. Within the domain of *Thinking and Feeling*, contributing factors may include a lack of perceived safety, efficacy, and trustworthiness regarding each vaccine as well as insufficient understanding of the diseases among pregnant women [15]. Among participants who expressed unwillingness to receive maternal vaccines, the most commonly cited reasons for hesitancy were uncertainty about fetal effects, concerns about adverse reactions, and insufficient information to make an informed decision. These findings suggest that, while overall intention to vaccinate may be relatively high, emotional and informational barriers remain substantial within the hesitant subgroup. Addressing these concerns through improved risk communication and tailored educational strategies may help further increase vaccine uptake. In terms of *Social Processes*, the delayed inclusion of these vaccines in Japanese guidelines may have led to insufficient recommendations from healthcare professionals, including physicians and midwives [7]. Regarding *Practical Issues*, while influenza vaccination is now standard practice in maternity care, pertussis vaccination is not commonly offered in such facilities in Japan. Furthermore, access to vaccines such as the recently approved RSV vaccine may remain limited [6,7,16]. This situation may be partly due to the absence of Tdap vaccine implementation in Japan. Although the DTaP vaccine—containing a different formulation of pertussis, diphtheria, and tetanus antigens—is currently approved for use in pregnant women in Japan, evidence regarding its immunogenicity and safety in the context of maternal immunization remains limited [7]. Therefore, maternal vaccination rates are expected to increase through the dissemination of accurate information, improved accessibility—such as reduced financial burden and broader availability at maternity facilities—and the accumulation of domestic data on vaccine immunogenicity and safety to enhance public trust.

Furthermore, this study showed that the factors associated with increased vaccination rates were prior knowledge of maternal immunization and the cost burden of vaccination. Previous studies have reported that a lack of knowledge regarding maternal immunization for pertussis is associated with a lower vaccine intention [11], which is consistent with the findings of this study. Additionally, a study on pertussis in the United Kingdom reported that although not statistically significant, the vaccine uptake rate during pregnancy differed by 69% in areas with low levels of deprivation compared with 52% in areas with high deprivation [17]. Although this study did not investigate deprivation levels, it showed that vaccination intention varied among pregnant women in Japan depending on the cost of vaccination. This may be partly due to the fact that in Japan, there is no public subsidy for maternal immunization with pertussis or RSV vaccines during pregnancy, and the recipient bears the entire cost.

Among respondents who did not intend to receive vaccination, “insufficient information to make a decision” and “uncertainty about vaccine efficacy” were more frequently cited for pertussis and RSV vaccines than for influenza. These differences likely reflect the historical development of vaccine recommendations for pregnant women in Japan. Since 2008, influenza vaccination has been addressed as a separate topic in obstetric guidelines, with its safety and effectiveness for newborns emphasized from 2011 onward [18,19]. As a result, influenza vaccination is widely practiced, and awareness among pregnant women is relatively high [20]. In contrast, pertussis vaccination was only mentioned for the first time in the 2023 edition, and the RSV vaccine, approved in 2024, has not yet been included in the guidelines [21]. These delays may explain the limited awareness and lower frequency of provider recommendations, contributing to greater hesitancy for the pertussis and RSV vaccines [6,7,8].

Based on our findings, two key strategies may help promote maternal immunization in Japan. First, given that prior knowledge of maternal immunization was more prevalent among those who expressed willingness to be vaccinated, the integration of educational interventions on vaccination into preconception care settings may represent a promising approach. However, this proposition should be interpreted with caution, as current evidence directly linking preconception education to increased vaccine uptake during pregnancy is limited. This concept aligns with international recommendations, such as those from FIGO [22]. In the context of Japan’s relatively high prevalence of fertility treatment [23], preconception care, especially in settings involving assisted reproductive technology (ART), may offer structured opportunities for anticipatory guidance on maternal immunization. Nevertheless, this hypothesis remains speculative and requires empirical validation in future research. Second, since the most frequently selected cost limit was JPY 3000, with most respondents willing to pay up to JPY 5000 for vaccination, financial support for vaccination programs may improve their uptake. In the United Kingdom and France, vaccination is currently subsidized by the government [16]; although it depends on the insurance plan, in the United States, it is often included as a covered service [24]. Although cost-effectiveness analyses of RSV vaccines have not been conducted in Japan, studies on influenza and pertussis vaccines have been conducted [25,26]. The cost-effectiveness of RSV vaccination according to epidemic season has been reported in other countries [27,28]. Although this depends on future epidemiological trends, financial subsidies may be a potential strategy from a social perspective in Japan, given that the absence of public financial support constitutes a well-recognized barrier to vaccine acceptance [29,30]. It is important to note that institutional barriers to maternal immunization in Japan are not limited to the lack of financial support systems. Notably, maternal immunization is not included in the official recommendation section of the Japanese clinical practice guidelines [16]. Due to this absence of strong recommendations, even if pregnant women are willing to be vaccinated, they may not receive active encouragement from healthcare providers in clinical settings, which may result in limited increases in actual vaccination coverage [31]. Although the present study did not assess whether participants received information about maternal immunization from healthcare providers during their current pregnancy, repeated counseling by healthcare professionals is known to be a key factor in promoting vaccine uptake [32]. Furthermore, there is evidence that reductions in institutional support may negatively impact vaccination coverage [33], suggesting that vaccine intention alone is insufficient to sustain behavioral change. Structural support and access must also be ensured. In recent years, chatbot-based interventions have been shown to enhance vaccine literacy and acceptance. Thus, the incorporation of digital tools—such as educational platforms and reminder systems—may be effective in translating high vaccination intent into actual uptake [34].

The strength of this study lies in its detailed clarification of vaccination awareness among pregnant women in Japan, which has not been reported in recent years, and the findings are important in terms of vaccination policies.

This study had some limitations. First, we recruited pregnant women from a wide range of facilities, from primary to tertiary obstetric ones; however, owing to the study design, we did not know the number of questionnaires collected at each facility. Furthermore, although the questionnaire was distributed to all pregnant women, the rate of response refusal was not measurable. Therefore, there may have been biases in the facilities and respondents, and participant bias cannot be excluded. Second, the study was conducted during the initial rollout phase of RSV vaccine availability in Japan, and future trends may differ. Third, as the questionnaire was developed de novo without formal validation or the use of established instruments, the reliability and validity of some items may be limited. Moreover, in the absence of cognitive pre-testing, respondents may have interpreted certain survey items inconsistently. Furthermore, the information sheet, which was provided to raise awareness, may have unintentionally influenced responses, introducing potential bias. Combined with the self-reported nature of the questionnaire, these factors may have introduced social desirability bias. Finally, this study was conducted exclusively at facilities located in urban settings. As the residential distribution of the respondents was not assessed, potential differences in health behaviors and vaccine literacy between urban and rural pregnant women could not be explored. As a result, the generalizability of the findings to rural or nationwide populations is limited, and different patterns may emerge in more diverse settings.

## 5. Conclusions

This study showed that awareness of maternal immunization among pregnant women in Japan remains low and the intention to be vaccinated is relatively high. Additionally, factors associated with increased vaccination rates are prior knowledge of maternal immunization and the cost burden of vaccination. To increase maternal immunization rates, integrating vaccine counseling into routine prenatal care may represent a feasible and effective strategy. Given the widespread preference for low-cost vaccination, implementing public financial support systems should also be considered to mitigate economic barriers. Future interventional studies are needed to evaluate the effectiveness of educational interventions, healthcare provider recommendations, and financial subsidies on actual vaccine uptake during pregnancy.

## Figures and Tables

**Table 1:** Background characteristics of pregnant respondents.

Variable	n (%)
**Age** **(year), median (IQR) ^a^**	33 (30–36)
**Gestational age at response** **^a^**	
up to 11 weeks	145 (28)
12 to 21 weeks	117 (22)
22 to 27 weeks	82 (16)
28 to 36 weeks	136 (26)
37 weeks or more	41 (7.9)
**Number of pregnancies** **, median (IQR)**	2 (1–3)
**Number of deliveries** **, median (IQR) ^b^**	0 (0–1)
**Conception methods** **^b^**	
Natural conception	327 (63)
Timed intercourse	51 (9.8)
Intrauterine insemination	12 (2.3)
IVF/ICSI	132 (25)
**Final educational attainment** **^b^**	
Junior high school	14 (1.7)
High school	65 (12)
Junior/vocational/technical college	141 (27)
University	261 (50)
Graduate school	41 (7.9)
**Experience in a healthcare occupation** **^b^**	
None	377 (72)
Hospital clerical work	24 (4.6)
Medical professional (e.g., nurse, pharmacist, lab tech)	109 (21)
Doctor	12 (2.3)
**Nationality**	
Japan	489 (93)
China	20 (3.9)
South Korea	3 (0.58)
Philippines	3 (0.58)
Vietnam	2 (0.38)
Thailand	2 (0.38)
Mongolia	2 (0.38)
Peru	1 (0.19)
Spain	1 (0.19)
**Prior knowledge of maternal immunization**	86 (16)

Note: Unless otherwise indicated, n = 523. Continuous variables are presented as medians with interquartile ranges (IQRs), and categorical variables as counts with corresponding percentages. Percentages are based on the number of respondents to each item. Abbreviations: IVF, In Vitro Fertilization; ICSI, Intracytoplasmic Sperm Injection. a, n = 521. b, n = 522.

**Table 2:** Preferred vaccination cost among pregnant women willing to receive each vaccine.

Preferred Cost (JPY)	Influenza (n = 351) n (%)	Pertussis (n = 303) n (%)	RSV (n = 305) n (%)	GBS (n = 364) n (%)
Willing to pay more than 50,000	16 (4.6)	14 (4.6)	17 (5.6)	13 (3.6)
Willing to pay up to 50,000	2 (0.6)	3 (1.0)	7 (2.3)	4 (1.1)
Willing to pay up to 30,000	6 (1.7)	5 (1.7)	6 (2.0)	4 (1.1)
Willing to pay up to 20,000	6 (1.7)	6 (2.0)	7 (2.3)	9 (2.5)
Willing to pay up to 10,000	34 (9.7)	34 (11)	37 (12)	38 (10)
Willing to pay up to 7000	7 (2.0)	11 (3.6)	10 (3.3)	8 (2.2)
Willing to pay up to 5000	106 (30)	90 (30)	78 (26)	116 (32)
Willing to pay up to 3000	160 (46)	122 (40)	129 (42)	152 (42)
Willing to pay up to 1000	14 (4.0)	18 (6.0)	14 (4.6)	20 (5.5)

Note: Values are presented as counts with corresponding percentages. Cost preferences were assessed only among participants who indicated willingness to receive each vaccine. “More than 50,000” refers to responses indicating no upper limit to payment. The number of respondents for each vaccine is shown in the column headers. Missing data were excluded (one missing for influenza; two missing for GBS). Abbreviations: JPY, Japanese Yen; RSV, respiratory syncytial virus; and GBS, group B streptococcus.

**Table 3:** Univariate logistic regression analysis of factors associated with willingness to receive influenza vaccination during pregnancy (n = 521).

Characteristic	Willing (n = 352)	Unwilling (n = 169)	OR (95% CI)
**Age (year)** **, median (IQR** **) ^a^**	34 (30–37)	33 (29–36.5)	N/A
**Gestational age** **, n (%)**			
<22 weeks	174 (49)	88 (52)	Ref
≥22 weeks	178 (51)	81 (48)	1.12 (0.779–1.62)
**Primiparity** **, n (%)**	187 (53)	98 (58)	0.821(0.567–1.19)
**IVF/ICSI, n (%)** **^b^**	96 (27)	35 (20)	1.44 (0.928–2.24)
**Education, n (%) ^b^**			
≤High school	42 (12)	37 (22)	Ref
≥Junior college ^c^	310 (88)	131 (78)	2.08 (1.28–3.39)
**Healthcare-related work (including clerical), n (%)** **^b^**	113 (32)	31 (18)	2.09 (1.33–3.28)
**Information source during pregnancy, n (%) ^b^** ^†^			
Doctor or midwife	326 (93)	142 (84)	2.48 (1.39–4.42)
Internet/SNS	188 (54)	90 (53)	1.01 (0.700–1.46)
Friend	55 (16)	38 (22)	0.641 (0.404–1.02)
**Prior knowledge of maternal immunization** **, n (%)**	74 (21)	12 (7.1)	3.48 (1.84–6.61)
**Prior knowledge of influenza infection, n (%)** **^b^**	343 (98)	159 (94)	2.70 (1.04–6.96)
**History of influenza infection (self or child)** **, n (%)**	295 (84)	133 (79)	1.40 (0.88–2.23)

Note: Continuous variables are presented as medians with interquartile ranges (IQRs); categorical variables are shown as counts with corresponding percentages. Percentages are based on the number of respondents to each item. † “Information source during pregnancy” allowed multiple selections; percentages are based on the number of respondents, not total responses. a: Two missing values were excluded from analysis for this item only; b: one missing value was excluded from analysis for this item only; and c: junior colleges include vocational and technical colleges. Abbreviations: IVF, In Vitro Fertilization; ICSI, Intracytoplasmic Sperm Injection; OR, Odds Ratio; CI, Confidence Interval; and SNS, Social Networking Service.

**Table 4:** Univariate logistic regression analysis of factors associated with willingness to receive pertussis vaccination during pregnancy (n = 518).

Characteristic	Willing (n = 303)	Unwilling (n = 215)	OR (95% CI)
**Age (year)** **, median (IQR** **) ^a^**	33 (30–36.5)	34 (30–37)	N/A
**Gestational age** **, n (%) ^a^**			
<22 weeks	143 (48)	115 (53)	Ref
≥22 weeks	158 (52)	100 (47)	1.27 (0.895–1.80)
**Primiparity** **, n (%)**	168 (55)	116 (54)	1.06 (0.748–1.51)
**IVF/ICSI, n (%)** **^b^**	78 (26)	52 (24)	1.09 (0.728–1.64)
**Education** **, n (%) ^b^**			
≤High school	36 (12)	42 (20)	Ref
≥Junior college ^c^	267 (88)	172 (80)	1.81 (1.16–2.94)
**Healthcare-related work (including clerical), n (%)** **^b^**	90 (30)	52 (24)	1.32 (0.884–1.96)
**Information source during pregnancy, n (%) ^b^** ^†^			
Doctor or midwife	279 (92)	186(87)	1.89 (1.06–3.37)
Internet/SNS	160 (53)	116 (54)	0.961 (0.677–1.37)
Friend	46 (15)	47 (22)	0.642 (0.409–1.01)
**Prior knowledge of maternal immunization** **, n (%)**	63 (21)	23 (11)	2.19 (1.31–3.66)
**Prior knowledge of pertussis infection, n (%)** **^b^**	254 (84)	168 (79)	1.42 (0.908–2.22)
**History of pertussis infection (self or child), n (%)** **^a^**	9 (3.0)	7 (3.3)	0.916 (0.336–2.50)

Note: Continuous variables are presented as medians with interquartile ranges (IQRs); categorical variables are shown as counts with corresponding percentages. Percentages are based on the number of respondents to each item. † “Information source during pregnancy” allowed multiple selections; percentages are based on the number of respondents, not total responses. a: Two missing values were excluded from analysis for this item only. Values in parentheses represent interquartile ranges; b: one missing value was excluded from analysis for this item only; and c: junior colleges include vocational and technical colleges. Abbreviations: IVF, In Vitro Fertilization; ICSI, Intracytoplasmic Sperm Injection; OR, Odds Ratio; CI, Confidence Interval; and SNS, Social Networking Service.

**Table 5:** Univariate logistic regression analysis of factors associated with willingness to receive RSV vaccination during pregnancy (n = 518).

Characteristic	Willing (n = 305)	Unwilling (n = 213)	OR (95% CI)
**Age (year)** **, median (IQR** **) ^a^**	33 (30–36)	33 (30–37)	N/A
**Gestational age, n (%)** **^a^**			
<22 weeks	150 (49)	108 (51)	Ref
≥22 weeks	153 (51)	105 (49)	1.05 (0.739–1.49)
**Primiparity** **, n (%)**	164 (54)	119 (56)	0.919 (0.646–1.31)
**IVF/ICSI, n (%)** **^b^**	81 (27)	49 (23)	1.22 (0.808–1.83)
**Education, n (%) ^b^**			
≤High school	36 (12)	41 (19)	Ref
≥Junior college ^c^	269 (88)	171 (81)	1.79 (1.11–2.92)
**Healthcare-related work (including clerical), n (%)** **^b^**	95 (31)	49 (23)	1.50 (1.01–2.25)
**Information source during pregnancy, n (%) ^b^** ^†^			
Doctor or midwife	279 (92)	186 (87)	1.62 (0.912–2.88)
Internet/SNS	159 (52)	116 (54)	0.917 (0.645–1.30)
Friend	49 (16)	44 (21)	0.738 (0.470–1.16)
**Prior knowledge of maternal immunization** **, n (%)**	64 (21)	22 (10)	2.30 (1.37–3.88)
**Prior knowledge of RSV infection** **, n (%)**	246 (81)	150 (70)	1.75 (1.16–2.64)
**History of RSV infection (self or child), n (%)** **^a^**	72 (24)	33 (15)	1.70 (1.08–2.68)

Note: Continuous variables are presented as medians with interquartile ranges (IQRs); categorical variables are shown as counts with corresponding percentages. Percentages are based on the number of respondents to each item. † “Information source during pregnancy” allowed multiple selections; percentages are based on the number of respondents, not total responses. a: Two missing values were excluded from analysis for this item only. Values in parentheses represent interquartile ranges; b: one missing value was excluded from analysis for this item only; and c: junior colleges include vocational and technical colleges. Abbreviations: IVF, In Vitro Fertilization; ICSI, Intracytoplasmic Sperm Injection; OR, Odds Ratio; CI, Confidence Interval; RSV, respiratory syncytial virus; and SNS, Social Networking Service.

**Table 6:** Univariate logistic regression analysis of factors associated with willingness to receive GBS vaccination during pregnancy (n = 519).

Characteristic	Willing (n = 366)	Unwilling (n = 153)	OR (95% CI)
**Age (year)** **, median (IQR** **) ^a^**	33 (30–36)	34 (29–37)	N/A
**Gestational age, n (%)** **^a^**			
<22 weeks	171 (47)	88 (58)	Ref
≥22 weeks	193 (53)	65 (42)	1.53 (1.04–2.24)
**Primiparity** **, n (%)**	194 (53)	90 (59)	0.790 (0.539–1.16)
**IVF/ICSI, n (%)** **^b^**	97 (27)	33 (22)	1.32 (0.839–2.06)
**Education, n (%) ^b^**			
≤High school	45 (12)	33 (22)	Ref
≥Junior college ^c^	321 (88)	119 (78)	1.98 (1.20–3.25)
**Healthcare-related work (including clerical), n (%)** **^b^**	114 (31)	31 (20)	1.79 (1.14–2.81)
**Information source during pregnancy, n (%) ^b^** ^†^			
Doctor or midwife	335 (92)	131(86)	1.73 (0.961–3.12)
Internet/SNS	199 (54)	77 (51)	1.16 (0.795–1.70)
Friend	63 (17)	30 (20)	0.846 (0.522–1.37)
**Prior knowledge of maternal immunization** **, n (%)**	70 (19)	15 (10)	2.18 (1.20–3.94)
**Prior knowledge of GBS infection, n (%)** **^b^**	108 (30)	25 (16)	2.13 (1.31–3.45)

Note: Continuous variables are presented as medians with interquartile ranges (IQRs); categorical variables are shown as counts with corresponding percentages. Percentages are based on the number of respondents to each item. † “Information source during pregnancy” allowed multiple selections; percentages are based on the number of respondents, not total responses. a: Two missing values were excluded from analysis for this item only. Values in parentheses represent interquartile ranges; b: one missing value was excluded from analysis for this item only; and c: junior colleges include vocational and technical colleges. Abbreviations: IVF, In Vitro Fertilization; ICSI, Intracytoplasmic Sperm Injection; OR, Odds Ratio; CI, Confidence Interval; GBS, group B streptococcus; and SNS, Social Networking Service.

## Data Availability

The datasets presented in this article are not readily available because they are part of an ongoing study. Requests to access the datasets were made by the corresponding author.

## Supplementary Materials

The following supporting information can be downloaded at https://www.mdpi.com/article/10.3390/vaccines13080779/s1, Question Items and Tables S1–S3. Table S1: Numbers of responses, missing values, and acceptance rates for each vaccine item. Abbreviations: RSV, respiratory syncytial virus; GBS, group B streptococcus.; Table S2: Reported reasons for unwillingness to receive maternal vaccines by vaccine type (influenza, pertussis, and RSV). Note: Among participants who expressed unwillingness to receive each vaccine, the numbers of respondents who answered the question on reasons for hesitancy were as follows: influenza, 143 of 169; pertussis, 193 of 215; and RSV, 195 of 213. Each column shows the number of respondents and the corresponding percentage among those who answered the question for each vaccine. Multiple responses were allowed; thus, totals may exceed 100%. Abbreviation: RSV, respiratory syncytial virus.; Table S3: Summary of variables significantly associated with willingness to receive each maternal vaccine in univariate logistic regression. Note: This summary table highlights variables that showed statistically significant positive associations with willingness to receive each vaccine based on univariate logistic regression analyses (Table 3, Table 4, Table 5 and Table 6). “+” indicates an Odds Ratio (OR) with a 95% Confidence Interval (CI) entirely above 1.0, suggesting a statistically significant positive association. “—” indicates no statistically significant association. Abbreviations: RSV, respiratory syncytial virus; GBS, group B streptococcus.

## Abbreviations

The following abbreviations are used in this manuscript:

CI	Confidence Interval
DTaP	diphtheria, tetanus, and acellular pertussis (vaccine)
FIGO	International Federation of Gynecology and Obstetrics
GBS	group B streptococcus
ICSI	Intracytoplasmic Sperm Injection
IVF	In Vitro Fertilization
JPY	Japanese Yen
OR	Odds Ratio
DT	respiratory syncytial virus
SNS	Social Networking Service
Tdap	tetanus, diphtheria, and acellular pertussis (vaccine)
