# Supplementary material for: Survey on Awareness and Attitudes Toward Maternal Immunization Against Influenza, Pertussis, Respiratory Syncytial Virus, and Group B Streptococcus Among Pregnant Women in Japan
Source: Vaccines (Basel). 2025 Jul 23;13(8):779. doi: 10.3390/vaccines13080779 (PMC12390194; doi:10.3390/vaccines13080779)
Supplement: Supplementary file 1 [file vaccines-13-00779-s001.zip › vaccines-3711278-supplementary.pdf]

Question Items
Maternal Vaccine Survey (Full English Translation)

Q1. Please indicate your age.
Q2. Please indicate your current gestational age (in weeks).
Q3. How many times have you been pregnant (including this pregnancy)?
Q4. How many times have you given birth (not including current pregnancy)?
Q5. What is your highest level of education completed?
Q6. What was the method of conception for this pregnancy?
Q7. Do you have any work experience in the medical field? If yes, please select your role. Multiple selections not allowed.
Doctor/Medical professional (e.g., nurse, pharmacist, lab tech)/Hospital clerical work/None.
Q8. Please indicate your nationality.
Q9. When obtaining information about vaccines during pregnancy, which sources do you consider important? (Multiple answers allowed.)
Q10. Do you know about maternal immunization?
Q11. Did you know about influenza, an infectious disease?
Q12. Have you or your child ever been infected with influenza?
Q13. Do you wish to be vaccinated against influenza during this pregnancy?
Q14. The vaccine is not covered by public health insurance and must be paid at your own expense. How much would you be willing to pay for the vaccine?
Q15. (If you do not intend to be vaccinated for influenza:) What are your reasons for not wishing to be vaccinated? (Multiple answers allowed. Select from the reasons listed in Figure 1.)
Q16. Did you know about pertussis, an infectious disease?
Q17. Have you or your child ever had pertussis?
Q18. Do you wish to be vaccinated against pertussis during this pregnancy?
Q19. The vaccine is not covered by public health insurance and must be paid at your own expense. How much would you be willing to pay for the vaccine?
Q20. (If you do not intend to be vaccinated for pertussis:) What are your reasons for not wishing to be vaccinated? (Multiple answers allowed. Select from the reasons listed in Figure 1.)
Q21. Did you know about RSV, an infectious disease?
Q22. Have you or your child ever been infected with RSV?
Q23. Do you wish to be vaccinated against RSV during this pregnancy?
Q24. The vaccine is not covered by public health insurance and must be paid at your own expense. How much would you be willing to pay for the vaccine?
Q25. (If you do not intend to be vaccinated for RSV:) What are your reasons for not wishing to be vaccinated? (Multiple answers allowed. Select from the reasons listed in Figure 1.)
Q26. Did you know about GBS, an infectious disease?
Q27. If the safety and efficacy for mothers and infants have been sufficiently verified,

would you like to receive the vaccine during pregnancy?
Q28. The vaccine may be at your own expense (public health insurance may not apply). If the safety and efficacy for mothers and infants have been sufficiently verified, how much would you be willing to pay for the vaccine?
Q29. In what conditions would you like to receive the vaccine? (Multiple answers allowed. Select from the reasons listed in Figure 2.)

Supplementary Table S1.
Numbers of responses, missing values, and acceptance rates for each vaccine item.

Vaccine	Number of respondents (n)	Willing to vaccinate (n)	Missing responses (n)	Acceptance (%)
Influenza	521	352	2	68
Pertussis	518	303	5	58
RSV	518	305	5	59
GBS	519	366	4	71

Abbreviations: RSV, respiratory syncytial virus; GBS, group B streptococcus.

Supplementary Table S2.
Reported reasons for unwillingness to receive maternal vaccines by vaccine type (influenza, pertussis, and RSV).

Reason for Hesitancy	Influenza (n = 143) n (%)	Pertussis (n = 193) n (%)	RSV (n = 195) n (%)
Uncertainty about the effects on the fetus	101 (71%)	106 (55%)	111 (57%)
Concern about adverse reactions	67 (47%)	77 (40%)	85 (44%)
Insufficient information to make an informed decision	46 (32%)	87 (45%)	96 (49%)
Uncertainty regarding vaccine efficacy	42 (29%)	71 (37%)	75 (38%)
Because the vaccine is not yet widely used in Japan	23 (16%)	42 (22%)	44 (23%)
The vaccination cost appears to be high	17 (12%)	29 (15%)	36 (18%)
I do not perceive a need for the vaccine	11 (7.7%)	13 (6.7%)	9 (4.6%)
I do not know where to receive the vaccination	4 (2.8%)	8 (4.1%)	9 (4.6%)
Other	21 (15%)	17 (8.8%)	12 (6.2%)

Note: Among participants who expressed unwillingness to receive each vaccine, the numbers of respondents who answered the question on reasons for hesitancy were as follows: influenza, 143 of 169; pertussis, 193 of 215; and RSV, 195 of 213. Each column shows the number of respondents and the corresponding percentage among those who answered the question for each vaccine. Multiple responses were allowed; thus, totals may exceed 100%.
Abbreviation: RSV, respiratory syncytial virus.

Supplementary Table S3.
Summary of variables significantly associated with willingness to receive each maternal vaccine in univariate logistic regression.

Variable	Influenza	Pertussis	RSV	GBS
Gestational age ≥22 wks	—	—	—	+
Higher education (≥ junior college)	+	+	+	+
Healthcare-related work	+	—	+	+
Doctor/midwife as info source	+	+	—	—
Prior knowledge of maternal immunization	+	+	+	+
Prior knowledge of infection	+	—	+	+

Note: This summary table highlights variables that showed statistically significant positive associations with willingness to receive each vaccine based on univariate logistic regression analyses (Tables 3–6).

"+" indicates an Odds Ratio (OR) with a 95% Confidence Interval (CI) entirely above 1.0, suggesting a statistically significant positive association.

"—" indicates no statistically significant association.

Abbreviations: RSV, respiratory syncytial virus; GBS, group B streptococcus.
